# The Gelatine Treatment of Aneurism

**Published:** 1901-08-31

**Authors:** 


### THE GELATINE TREATMENT OF ANEURISM.

The two unfortunate cases in which death has followed the injection of a solution of gelatine for the cure of aneurism, at Guy's Hospital, serve to illustrate the risks that must attend all forms of treatment which involve the introduction of foreign substances into the blood or lymph stream. It appears that the two men in question were undergoing treatment for aneurism and had already had in the one case six or seven, and in the other 10 injections, when death resulted, as the consequence apparently of microbic infection which had by some means obtained access to the solution employed. We may say at once that the rarity of such accidents is good proof of the extreme care that is habitually taken in the preparation of the various forms of antitoxic serum which are now in constant use in the treatment of disease, while their occasional occurrence (as, for example, the series of cases which lately took place in Italy, where tetanus followed the injection of antidiphtheritic serum) shows that the danger is no fanciful one, and that but for the precautions taken all these injection treatments would be quickly discredited. The matter is of all the more importance in view of recent developments in this form of medication. In the treatment of shock and of hæmorrhage the injection of saline solution, either into the veins or into the subcutaneous tissues, has become a routine proceeding and a large number of patients have been snatched by it from imminent death. In various toxic conditions, again, such as puerperal eclampsia, this process has in some hands been attended with brilliant results, but we must never forget the possibility of infection, either in the solution or the syringe, or much more probably in the skin of the patient, the extempore sterilisation of which is by no means always an easy task in the emergencies in which these methods are mostly employed. Of late, again, a good deal has been heard about the introduction of antiseptics into the blood, such as formalin in the treatment of tuberculosis, and protargol in that of pneumonia; so that the occurrence of these two cases to which we have alluded may well serve as a warning to the effect that methods of this kind are not to be lightly undertaken. The puzzle and the difficulty in regard to these cases is, however, that they were not lightly undertaken; the greatest care seems to have been given to the sterilisation of the gelatine solution, and it is by no means easy to say at what point the infection arose.

In regard to this method of treating aneurism, it is now about six years since it was found that the injection of gelatine into the veins of a dog made the blood more coagulable, and four years ago Lancereaux and Paulesco reported in the *Gazette des Hôpitaux* a case of aortic aneurism which they had treated by the subcutaneous injection of a 1.0 per cent. solution of gelatine in a 0.1 per cent. solution of sodium chloride. Of this solution 50 c.c. were at first injected, then the amount was increased to 150 c.c. Twelve of these injections were given, and considerable improvement followed. Since then this treatment has been employed in a considerable number of cases. In 1898 Lancereaux, writing on the subject, described his methods. The solution he used consisted of white gelatine 4.5 grammes, sodium chloride solution (7 per 1,000) 200 c.c. This solution was sterilised at 120° C. He advised that several samples should be prepared, and that those which became turbid or did not solidify should be rejected. All the apparatus to be employed for the injection was to be thoroughly sterilised, the site of injection, which was usually the buttock, was to be purified, and the point of the needle was to be driven deeply into the tissues. The total amount of the injection should be got in in about 15 minutes, and the process was to be repeated every six or eight days. In the *Journal of the American Medical Association* for January, 1900, there is an article by Futcher in which he reports nine cases of aortic aneurism so treated. He gave his injections more frequently than Lancereaux had recommended, but his results showed that in no case was the aneurism cured, although in one it had diminished considerably in size, and in seven there had been appreciable diminution of the pressure symptoms. In some of his cases the injections had been followed by a chill and pyrexia, so that it is not impossible that the solution may have not been absolutely pure. In the *Gazz. degli Osped.* for February, 1900, Geraldini records four cases of aortic aneurism treated by the subcutaneous injection of a 2 per cent. solution of gelatine. A large number (40 to 60) injections were given in these cases. So far as the subjective symptoms go the results were good, and there was lessening in the size and pulsation of the tumour. This year K. Barth (*Münch. med. Woch.*, April 2), describes a case of aneurism of the aorta treated in this way. Twelve injections completed the first course and later on another course was administered, but other means also were employed. The result was good as regards the subjective, but much less marked in regard to the objective symptoms. Thus summing up the matter there is good ground for believing that by the injection of sterile solution of gelatine the coagulability of the blood is increased and that the cure of an aneurism is facilitated. It must be confessed, however, that this method of treatment is still within the field of experimental medicine.

